# Cracks in the JD-R model? The failure of strengths use, job crafting, and home-work spillover to support wellbeing during COVID-19

**DOI:** 10.3389/fpsyg.2025.1532083

**Published:** 2025-06-12

**Authors:** Llewellyn Ellardus van Zyl, Menno A. Cornelisse, Pascale Le Blanc, Sebastiaan Rothmann

**Affiliations:** ^1^Optentia Research Unit, North-West University (Vaal Triangle Campus), Vanderbijlpark, South Africa; ^2^Psynalytics : AI Powered People Analytics, Eindhoven, Netherlands; ^3^Technical University Eindhoven, Eindhoven, Netherlands

**Keywords:** job demands, job resources, job crafting, strengths use, work-home interaction, psychological wellbeing, JDR approach

## Abstract

Drawing from the Job Demands-Resources (JD-R) model, this study examined the relationship between job characteristics, work-home interference, motivation, and psychological wellbeing during the COVID-19 lockdown. Specifically, it explored whether individual-level strategies such as strengths use, job crafting, and home-work regulatory factors (i.e., positive and negative home-work spillover) moderated these relationships. A cross-sectional survey of 522 participants was conducted during the lockdown. Structural equation modelling, mediation, and moderation analyses tested the proposed relationships. Results showed that work overload, organizational support, and job security were significantly associated with both negative and positive work-home interference, while growth opportunities and advancement were not. Positive and negative work-home interference and motivation were directly linked to psychological wellbeing, although only positive work-home interference was associated with motivation. Further, strengths use and job crafting moderated was only found to moderate the relationship between job security and negative work-home interaction. Finally, home regulatory practices may not be helpful in explaining how job characteristics affect the work-home relationship. The findings suggest that during crises, the JD-R model falls short in accounting for the complex interaction between job characteristics and employee outcomes. While structural factors like work overload, organizational support, and job security remain central, individual strategies and home-regulatory practices had limited impact. These insights challenge assumptions about the JD-R model's “universal applicability” and the presumed effectiveness of personalized coping strategies during systemic disruption. It also exposes a deeper limitation of the JD-R model: its implicit tendency to pathologize the employee by placing the burden of wellbeing on individuals rather than addressing the systemic conditions that shape it. In times of crisis, the onus should not be on employees to adapt, but rather on organizations to create environments that support work-life balance and sustainable wellbeing.

## Introduction

The COVID-19 lockdowns forced most of the working population into remote working, which blurred the boundaries between work- and home life. Since remote work was not widely employed before the pandemic (Dubey and Tripathi, 2020), and most organizations were ill-prepared to support this practice (Yang et al., 2023), the COVID-19 lockdowns led to increasing reports of work-home interference (Schieman et al., 2021; Wood et al., 2022). Work-home interference refers to the extent that emotional, cognitive, mental or physical demands of work and work-roles positively or negatively affect personal/home life and interests (Brough et al., 2020). Work-home interference is experienced negatively when there is an incompatibility between work- and life roles due to high job demands (i.e., Negative Work-Home Interference: NWHI), and positively when positive experiences from work make it easier to engage in home/life-related tasks due to an abundance of resources (i.e., Positive Work-Home Interference: PWHI; Montgomery et al., 2003). Those who reported high levels of work-home conflict during the pandemic argued that it prevented them from focusing on important aspects of their personal lives, that there was a lack of energy to invest in social relationships, and that their work-roles prevented them from attending to home-related tasks or roles (Schieman et al., 2021). This increased prevalence of work-home interference is argued to be a result of the sudden and significant increase in job demands, coupled with limited access to job resources caused by the sudden shift to forced remote work (Koekemoer et al., 2021; Parham and Rauf, 2020). This imbalance in job demands/resources and increased work-home interference led to various unfavorable individual and organizational outcomes. For the individual, this resulted in more reports of common mental health problems (stress, depression, anxiety), emotional distress, burnout, substance abuse disorders, sleep disorders, and even post-traumatic stress (Giorgi et al., 2020; Macaron et al., 2023; Soares et al., 2022; Van Zyl et al., 2021a) as well as lower levels of motivation, and overall psychological wellbeing (cf. Bakker et al., 2023). For organizations, this resulted in lower levels of productivity, performance, and financial turnover, as well as increased staff attrition and -turnover rates (Bakker et al., 2023).

Although there is a large body of literature confirming the negative effects of work-home interference during the pandemic, some studies have suggested that forced remote work has had positive effects on employees' wellbeing and work-related outcomes (Harju et al., 2021; Kitagawa et al., 2021; Meyer et al., 2021; Tušl et al., 2021). Various studies during the pandemic have shown that for some employees, work engagement, motivation, and psychological wellbeing increased (Chambel et al., 2022; Harju et al., 2021; Kitagawa et al., 2021; Miawati et al., 2021; Meyer et al., 2021; Tušl et al., 2021; Zhang et al., 2021). For example, Mäkikangas et al. (2022) showed that during the first wave of the pandemic, 75% of their sample reported above-average to high levels of work engagement. Similar trends were found for work motivation (cf. Baladraf and Pogo, 2022; Ilea et al., 2023; Kaya et al., 2022). In a general Danish sample, Sønderskov et al. (2020) found that psychological wellbeing increased significantly during the first and second COVID-19 waves. Additionally, O'Connor et al. (2021) found that employees over the age of 30 in the UK reported higher levels of positive wellbeing throughout the pandemic, regardless of their ethnic background. Further, Harju et al. (2021) found that a substantial proportion of employees reported moderate to high levels of flourishing. There is thus a significant contradiction within the pandemic-related literature as to the effect forced remote work had on individuals.

### The job demands and resources framework: a work-home interface perspective

The Job Demands and Resources theory provides an interesting lens through which to interpret these contradictory findings (JDR: Demerouti and Bakker, 2022). According to Demerouti and Bakker (2022), work contexts play an imperative role in understanding how job characteristics affect individual wellbeing and organizational performance in times of crisis. Job characteristics are categorized as a dynamic interaction between job demands and job resources, affecting the extent to which work can interfere with life (Montgomery et al., 2003). According to Van Zyl et al. (2022, p. 2) *job demands* “refer to the physical-, social-, or organizational aspects of a job that require enduring effort and are associated with lasting physical/psychological costs.” The job demand most frequently reported during the pandemic was work overload (i.e., the inability of an individual to cope with the pace and amount of work; Bakker et al., 2023). *Job resources*, on the other hand, pertain to the “physical, social, or organizational aspects of the job that are important to (a) facilitate organizational goals, (b) reduce demands, strain, and the physical/psychological costs of work and (c) stimulate personal growth and development” (Van Zyl et al., 2022, p. 2). This includes aspects such as organizational support (the relationship with supervisors and colleagues, clarity in work-roles, participation, and contact possibilities), job security (certainty and clarity about one's future in the organization), advancement (promotion and career progression opportunities), and growth opportunities (variety in work, opportunities to learn/develop and autonomy at work; Jackson and Rothmann, 2005).

These job characteristics affect work-home interference and psychological wellbeing through a health impairment- and a motivational process (Demerouti and Bakker, 2022). The *health impairment process* is activated when high job demands and low resources gradually drain the mental energies of individuals, resulting in poor wellbeing and performance over time (Van Zyl, 2025, Van Zyl et al., 2024c, 2021a). During forced remote work, the resultantly high job demands increased employees' stress and strain responses, which in turn results in work more negatively interfering with home/personal life and ultimately negatively affecting their motivation and psychological wellbeing (Demerouti and Bakker, 2022). In contrast, the *motivational process* involves the presence of an abundance of resources that leads to more positive work-home spill-over, which in turn leads to greater motivation, and psychological wellbeing (Demerouti and Bakker, 2022). Job resources also buffer against high job demands' negative effect on important individual- and organizational outcomes (Bakker et al., 2023). In other words, when employees had access to abundant resources (e.g., increased autonomy) during the pandemic, they may have had more positive work experiences which spilled over into home life. This may have increased their motivation to perform and thus led to higher reports of psychological wellbeing (Bakker et al., 2023).

### The role of strength use, job crafting, and home-work interference

According to Van Zyl et al. (2023) the role these job characteristics played in relation to employees' work-home interference, motivation, and psychological wellbeing during the pandemic was offset by several factors. Those who could deploy their personal resources (e.g., strengths use), who engaged in proactive individual regulatory practices (e.g., job crafting), and who experienced positive outcomes due to effectively managing their proactive- and destructive home regulatory factors (e.g., positive- and negative home-work interference), were better equipped to manage the challenges of forced remote work and its impact on work-home interaction and wellbeing (Bakker et al., 2023; Van Zyl et al., 2024d).

*Personal resources* within the JDR framework are positioned as the individual characteristics employees use to enhance their perceptive control over the work environment, to improve person-environment fit and to increase resilience at work (Bakker and Demerouti, 2017). Personal resources help to buffer against the negative effect of job demands and enhance the positive effects of job resources on individual and organizational outcomes (Bakker et al., 2014). An important personal resource to consider is an employee's capacity to identify and apply their personal strengths in the execution of their work-related tasks (i.e., strengths use: Van Zyl et al., 2021b, 2024a). Strengths refer to inherent positive psychological traits that employees are naturally good at, and, when actively used, lead to more optimal functioning and wellbeing (Van Zyl et al., 2024a,b). When employees can use their strengths more actively at work, it may reduce the effect of how high pandemic-related job demands tend to increase negative work-home spillover, but it could also strengthen or the enhance effect of perceptions of high job resources tend to create more positive work-home spill over (Brough et al., 2020).

Another strategy individuals employed during the pandemic was the activation of *proactive individual regulatory practices* (Demerouti and Bakker, 2022). These are individual-level strategies individuals employed to manage, change or control the work environment and how this affects functioning in various life domains during the pandemic (Demerouti and Bakker, 2022). These strategies increase their available job resources, and challenging demands as well as decrease hindering demands (i.e., job crafting: Demerouti and Bakker, 2022). Job crafting refers to the proactive and adaptive changes individuals make to their work-related tasks, social relationships and their perceptions of work in order to align work with their personal preferences, goals, and strengths (Van Zyl et al., 2023). Demerouti and Bakker (2022) argued that job crafting helps individuals in coping with the challenges brought on by forced remote working and provides a means to manage the work-home interface more effectively. Specifically, they propose that job-crafting buffers the unfavorable impact of pandemic-related job demands on health and boosts the positive impact of resources on motivation and wellbeing.

Finally, Demerouti and Bakker (2022) highlighted the importance of the role outcomes of effectively managing *proactive- and destructive home regulatory factors* (e.g., positive- and negative home-work interference) play in shaping the work-home interaction during the pandemic and in managing the effect job characteristics (demands/resources) have on motivation and wellbeing. *Proactive home regulatory* factors are those elements from one's home life that are activated to help buffer against the effects of high job demands on the work-home interface and wellbeing (e.g., the egalitarian division of labor at home and boundary management; Demerouti and Bakker, 2022). More specifically, when these strategies are effectively actioned, it helps individuals experience more positive experiences at home, spilling over into work (Positive Home-Work Interference: PHWI). *Destructive home regulatory factors* relate to those elements of home life, such as spousal undermining behaviors or gender division of labor, that increase negative home-work spill-over (Negative Home-Work Interference: NWHI). Therefore, the outcomes of proactive- and destructive home regulatory factors such as positive- or negative home-work experiences, may play a buffering role in how job demands and resources affect the work-home relationship, motivation, and wellbeing (Demerouti and Bakker, 2022; Meyer et al., 2021).

### The current study

**Figure 1:**
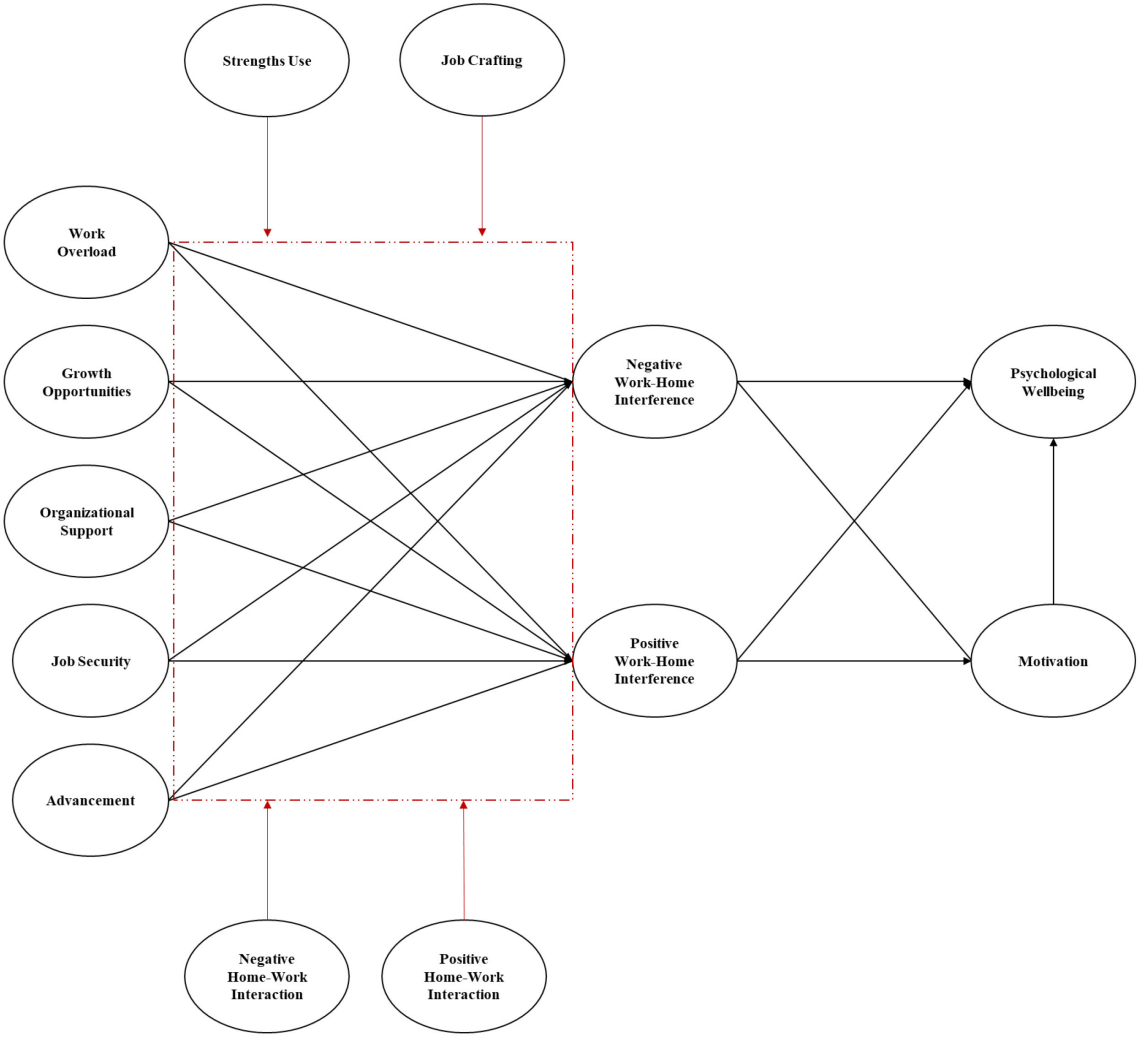
Conceptual model.

It is thus important to not only understand how the relationship between job characteristics, work-home interference, motivation, and psychological wellbeing played out during the pandemic, but it is essential to investigate how personal resources, individual regulatory practices and the outcomes of home regulatory strategies influence this relationship. As such, this paper aims to examine the role of job characteristics (demands/resources), work-home interference, motivation, and psychological wellbeing during the COVID-19 lockdowns, and to determine whether work-home interference and motivation indirectly affect the relationship between job characteristics and psychological wellbeing. Drawing from the JDR perspective, the study aims to provide insights into the mechanisms through which personal resources (such as strengths use) and proactive individual regulatory practices (such as job crafting) can help employees effectively navigate the challenges of remote work during times of crisis. It further aims to determine how positive- and negative-home spill-over (as outcomes of proactive and destructive home regulatory factors) can buffer or exacerbate job characteristics' impact on the work-home interaction and psychological wellbeing. The conceptual framework of this study is presented in Figure 1.

These general objectives translate into the following specific hypotheses:

***Direct relationships***

H_1_: Job demands (work overload) positively relate to NWHI and negatively relate to positive work-home interference PWHI.

H_2_: Job resources (organizational support, job security, growth opportunities, advancement) negatively relate to NWHI and positively relate to PWHI.

H_3_: NWHI is negatively related to psychological wellbeing and motivation.

H_4_: PWHI is positively related to psychological wellbeing and motivation.

H_5_: Motivation is positively related to psychological wellbeing.

***Indirect relationships* *(mediation)***

H_6_: Positive and negative work-home interference, as well as motivation, sequentially mediate the relationship between job characteristics (demands/resources) and psychological wellbeing.

***Moderation***

H_7_: Strengths use and job crafting moderate the relationship between job characteristics and work-home interference, such that higher levels of strengths use and job crafting weaken the negative impact of demands and enhance the positive impact of resources.

H_8_: Positive and negative home-work interference moderate the relationship between job characteristics and work-home interference, such that home-work interference amplifies the corresponding form of work-home interference.

## Methodology

### Research approach

A quantitative cross-sectional survey-based research design was employed to investigate the relationship between the focal variables of this study. The design allowed for data to be obtained from a large sample of employees at a single time point during the COVID-19 lockdowns. Surveys were in English and distributed electronically through the researchers' professional and social media networks.^1^

### Participants and procedure

A power analysis using G-Power (Faul et al., 2009: alpha = 0.05, power = 0.90, *d* = 0.30), pwrSEM (Wang and Rhemtulla, 2021: alpha = 0.05, Simulations: 10,000) and equation Westland's (2010: *d* = 0.30, power = 0.90, *p* < 0.01) was used to determine appropriate sample size. Cumulatively, the results indicated that a minimum sample size of 95 is required to fit the model structure and 310 to detect the desired effect. To compensate for potential sampling error, a non-probability-based, convenience sampling strategy was used to draw 634 initial participants for this study. The validity of the responses and data quality were assessed by implementing several attention checks (e.g., “Please rate item 13 on the scale as Completely Disagree”; “Write the word sky in the textbox and rate it as Absolutely Agree”; cf. Abbey and Meloy, 2017) and a *post-hoc* analysis of response patterns, responses consistency, and completion times (cf. Buchanan and Scofield, 2018). Participants who did not accurately respond to the attention checks and were found to answer questions randomly or deviated significantly from the median response time were excluded from the final dataset.

Table 1 provides a descriptive summary of the demographic characteristics of the final sampled population (*N* = 522). Most participants in this study were English-speaking (32.4%) females (51.7%) from Europe (51.9%) between the ages of 26 and 35 (28.6%) with a PhD or equivalent degree (31.2%). Further, most participants lived with one or two other people in their household (54.6%) and did not have children (61.9%). The majority of the participants were employed full-time (76.4%) within the higher education and scientific research industry (35.4%) and were forced to work from home during the COVID-19 pandemic (90.8%). Prior to the COVID-19 pandemic, most participants did not engage in hybrid working arrangements (46.5%).

**Table 1:** Characteristics of the participants (*N* = 522).

**Item**	**Category**	**Frequency (*f*)**	**Percentage (%)**
Gender	Male	250	47.9
	Female	270	51.7
	Unknown	2	0.4
Age	18–25 years	54	10.3
	26–35 years	149	28.6
	36–45 years	147	28.1
	46–55 years	93	17.9
	56–65 years	62	11.8
	66 years and older	17	3.3
Highest qualification	High school	48	9.2
	Diploma	88	16.9
	Bachelors degree	70	13.4
	Masters degree	147	28.2
	Ph.D. or equivalent	163	31.2
	Prefer not to say	6	1.1
Home language	African	71	13.6
	Dutch	139	26.6
	English	169	32.4
	German	61	11.7
	Other	82	15.8
Living with people (18+)	Alone	74	14.2
	1–2	285	54.6
	3 or more	163	31.2
Number of children (18–)	No children	322	61.9
	1 or more children	198	38.1
Employment status	Working full time	399	76.4
	Working part time	80	15.3
	Retired	9	1.7
	Unemployed	5	1
	Intern	29	5.6
Forced to work from home during COVID-19	Yes	474	90.8
	No	48	9.2
Prior to COVID working from home	Yes	99	19.0
	No	243	46.5
	Partially	180	34.5
Country of residence	European	271	51.9
	Australia	3	0.6
	North America	45	8.6
	United Kingdom	72	13.8
	South America	6	1.1
	Asia	7	1.3
	Africa	118	22.6

### Measures

The following self-report instruments were used to assess the focal factors and moderating variables for the study:

#### Focal variables

A shortened version of the *Job Demands- and Resources Scale* (JDRS: Jackson and Rothmann, 2005) was used to measure participants' perceived job demands and job resources. The instrument consists of 19 items which are rated on a five-point Likert-type scale ranging from 1 (Never) to 5 (Always). The scale measures five factors: (a) work overload (e.g., “Do you work under time pressure?”), (b) growth opportunities (e.g., “Does your job offer you opportunities for personal growth and development?”), (c) organizational support (e.g., “Can you discuss work problems with your direct supervisor?”), (d) job security (e.g., “Do you need to have more security to feel that you will still be working in one year's time?”), and (e) advancement (e.g., “Does your job offer you the possibility to progress financially?”). Acceptable levels of internal consistency and reliability have been reported in previous studies with Cronbach Alphas ranging from 0.76 to 0.92 on the various subscales (Rothmann et al., 2006; Rothmann and Jordaan, 2006).

The positive and negative work-home interaction subscales of the shortened version of the “*Survey Work-Home Interaction—NijmeGen”* scale (SWING: Geurts et al., 2005) were used to measure work-home interaction. The eight-item self-report subscale measures two constructs on a five-point Likert scale ranging from 1 (Never) to 5 (Always): (1) Negative Work-Home Interference (NWHI: “You have to work so hard that you do not have time for any of your hobbies?”) and (2) Positive Work-Home Interference (PWHI: “You are better able to keep appointments at home because your job requires this as well?”). Geurts et al. (2005) reported acceptable levels of internal consistency with Cronbach alphas exceeding the suggested cut-off scores on the various subscales (α scores: NWHI = 0.85; PWHI = 0.72).

The intrinsic motivation subscale of the *Multidimensional Work Motivation Scale* (MWMS: Gagné et al., 2015) was used to assess participants' self-reported job motivation. The sub-scale measures intrinsic motivation with three items based on a seven-point Likert-type scale ranging from 1 (Not at All) to 7 (Completely). It is comprised of items such as “Because I have fun doing my job.” The scale showed both acceptable data model fit and a Cronbach Alpha of 0.80 in a previous study, indicating good reliability levels (Gagné et al., 2015).

The psychological wellbeing (PWB) sub-scale of the *Mental Health Continuum-Short Form* (MHC-SF; Keyes et al., 2008) was used to measure self-reported psychological wellbeing. The sub-scale consists of six self-report items which are rated on a six-point Likert scale ranging from 1 (Never) to 6 (Every Day). The MHC-SF requests participants to reflect upon the past month's experiences and indicates the extent to which they experienced their PWB with items such as “That you have experiences that challenge you to grow and become a better person.” The PWB subscale has shown to be highly reliable, with Cronbach Alphas and Mc Donald's omegas ranging from 0.89 to 0.95 in various studies (Lamers et al., 2011; Van Zyl and Ten Klooster, 2022).

#### Moderating factors

The *Strength Use Scale* (SUS: Govindji and Linley, 2007) was used to measure the extent to which individuals actively use their strengths in daily life. The fourteen-item self-report measure is rated on a seven-point Likert scale ranging from 1 (Strongly Disagree) to 7 (Strongly Agree). The instrument measures strengths use as a function of two constructs: (a) active strengths use (“I use my strengths everyday”) and (b) strengths-based affinity (“I pursue goals and activities that are aligned to my strengths”). The SUS showed acceptable levels of internal consistency with point composite reliability, Mc Donald's omegas and Cronbach's alphas ranging from 0.86 to 0.93 on the various subscales over time (Van Zyl et al., 2021a).

The *Job Crafting Questionnaire* (JCQ: Tims et al., 2022) was employed to measure the job crafting ability of employees. The instrument comprises 21 items rated on a five-point Likert-type scale ranging from 1 (Totally Disagree) to 5 (Totally Agree). From this perspective, job crafting is seen as a function of four inter-related factors: (1) increasing structural job resources (“I try to learn new things at work”), (2) increasing social job resources (“I ask others for feedback on my job performance”), (3) increasing challenging job demands (“When an interesting project comes along, I offer myself proactively as project co-worker”), and (4) decreasing hindering job demands (“I try to ensure that my work is emotionally less intense”). The instrument has shown high levels of internal consistency with Cronbach Alphas above the suggested ranges in previous studies (Tims et al., 2022; Topa and Aranda-Carmena, 2022).

The positive and negative home-work interaction subscales of the shortened version of the “*Survey Work-Home Interaction—NijmeGen”* scale (SWING: Geurts et al., 2005) were used to measure home-work interaction. The eight-item subscale measures two constructs on a five-point Likert scale ranging from 1 (Never) to 5 (Always): (1) Negative Home-Work Interference (NHWI: “Problems with your spouse/family/friends affect your job performance?”) and (2) Positive Home-Work Interference (PHWI: “You have greater self-confidence at work because you have your home life well organized?”). Geurts et al. (2005) reported acceptable levels of internal consistency with Cronbach alphas exceeding the suggested cut-off scores on the various subscales (α scores: NHWI = 0.72; PHWI = 0.78).

### Statistical analysis

JASP v 0.17.1 (JASP, 2021), SPSS v. 28 (IBM Corp, 2020) and Mplus v 8.8 (Muthén and Muthén, 2023) were used to analyze the data. The primary analysis was conducted through the structural equation modeling framework with the maximum likelihood estimation method. Missing data was managed through the full maximum likelihood estimation approach.

*First*, the descriptive statistics, upper- and lower-bound reliability estimates, and Pearson correlation coefficients were estimated to test assumptions and explore the data. The results are summarized in Appendix A.

*Second*, a series of theoretically informed competing measurement models were estimated and compared to determine the best-fitting model for the data. Both traditional data-model fit criteria (cf. Table 2) and indicators of measurement quality were used to evaluate models (Van Zyl and Ten Klooster, 2022). Measurement quality was established by inspecting various parameter estimates (standardized factor loadings λ > 0.30; item uniqueness > 0.1 but <0.9; no cross-loadings; Van Zyl and Ten Klooster, 2022). Only the comparatively best-fitting measurement model was retained and converted to a structural model to determine the linear relationship between latent factors (Muthén et al., 2017). The same model fit, and measurement quality indicators were used to evaluate the data-model fit. The significance of the direct relationships was set at *p* < 0.05.

**Table 2:** Model fit statistics.

**Fit indices**	**Cut-off criterion**	**Sensitive to *N***	**Penalty for model complexity**
**Absolute fit indices**			
Chi-square (χ^2^)	Lowest comparative value between measurement model	Yes	No
	Non-significant chi-square (*p* > 0.01)		
**Approximate fit indices**			
Root-means-square error of approximation (RMSEA)	0.06–0.08 (acceptable); 0.01–0.05 (excellent)	No	Yes
	Non-significant RMSEA (*p* > 0.01)		
	90% confidence interval range should not include zero		
Standardized root mean square residual (SRMR)	0.06–0.08 (acceptable); 0.01–0.05 (excellent)	Yes	No
**Incremental fit indices**			
Comparative fit index (CFI)	0.90–0.95 (acceptable Fit); 0.96–0.99 (excellent)	No	No
Tucker-Lewis index (TLI)	0.90–0.95 (acceptable fit); 0.96–0.99 (excellent)	No	Yes
Akaike information criterion (AIC)	Lowest value in comparative measurement models	Yes	Yes
Bayes information criterion (BIC)	Lowest value in comparative measurement models	Yes	Yes

Adapted from Van Zyl and Ten Klooster (2022).

*Third*, a sequential or “serial” mediation model was specified to estimate the overall- and specific indirect effect NWHI and/or PWHI had on the relationship between job characteristics (demands/resources), motivation and psychological wellbeing. Similarly, the specific indirect effect of motivation on the relationship between PWHI and psychological wellbeing was estimated. The bias-corrected bootstrapping method was used with 50 000 bootstraps to impute the indirect effect estimate at the 95% confidence interval range. To establish serial mediation, the standardized indirect effect estimate of the overall model should be significant (*p* < 0.05), and the confidence interval range should not include zero (Wang and Wang, 2020).

*Finally*, a moderation model with the direct effects of job characteristics (demands/resources) on NWHI and PWHI being moderated by strengths use, job crafting, and NHWI/PHWI^2^ was estimated using Hayes' (2017) PROCESS v.4 macro in SPSS v. 27 (IBM Corp, 2020). The factor scores of the best-fitting measurement model were computed, saved and used for the moderation estimation (DiStefano et al., 2009). An interaction term was created by the product of the independent variables and the intended moderator. Evidence of moderation could be inferred if the interaction term is significantly related to the dependent variable (*p* < 0.05) and if the confidence interval range does not include zero (Hayes, 2017).

## Results

### Comparing competing measurement models

A series of theoretically informed competing confirmatory factor analytical measurement models were estimated and compared to find the best-fitting model for the data. Measured items were treated as indicators for first-order latent factors, where items were estimated to load directly onto their a priori factors. Only focal factors of the model were estimated. No items were removed or parceled.

In total, nine measurement models were tested:

- **Model 0**: First-order factor models were specified for overall job characteristics, work-home interference, motivation, and psychological wellbeing.
- **Model 1**: First-order factorial models were specified for job demands, job resources, work-home interference, motivation, and psychological wellbeing.
- **Model 2**: First-order factorial models were specified for work overload, organizational support, job security, growth opportunities, advancement, negative work-home interaction, positive work-home interaction, motivation, and psychological wellbeing.
- **Model 3**: A second-order factor model was estimated for overall job resources comprised of four first-order factors (organizational support, job security, growth opportunities, and advancement). First-order factors were specified for work overload, NWHI, PWHI, motivation, and psychological wellbeing.
- **Model 4**: A second-order factor model was estimated for overall work-home interference which was comprised of two first-order factors (NWHI and PWHI). First-order factors were specified for work overload, organizational support, job security, growth opportunities, advancement, motivation, and psychological wellbeing.
- **Model 5**: Two second-order factorial models were specified for job resources (comprised of organizational support, job security, growth opportunities, and advancement) and overall work-life interaction (comprised of NWHI, PWHI). First-order factors were specified for work overload, motivation, and psychological wellbeing.
- **Model 6**: A third-order factorial model for job characteristics was specified that comprised of a second-order factorial model for job resources (organizational support, job security, growth opportunities, advancement) and a first-order factor model for work overload. First-order factors were specified for NWHI, PWHI, motivation, and psychological wellbeing.
- **Model 7**: A second-order factorial model was specified for overall job characteristics (work overload, organizational support, job security, growth opportunities, advancement). First-order factorial models were specified for NWHI, PWHI, motivation, and psychological wellbeing.
- **Model 8**: Two second-order factorial model was specified for overall job characteristics (work overload, organizational support, job security, growth opportunities, advancement) and overall work-home interaction (NWHI, PWHI). First-order factorial models were specified for motivation, and psychological wellbeing.

Table 3 summarizes the model fit statistics for the nine competing measurement models. In line with the hypothesized model (Figure 1), the results showed that Model 2 fitted the data significantly better than the other models [**Model 2:** $\chi_{\left(522\right)}^{2}$ = 931.88; *df* = 491; CFI = 0.96; TLI = 0.95; RMSEA = 0.04 [0.036–0.046]; *p* > 0.05; SRMR = 0.05; AIC = 42650.58; BIC = 43238.14; aBIC = 42800.10]. Model 2 showed a comparatively better fit than all other models with a lower χ^2^, RMSEA, SRMR, AIC, and BIC values as well as higher CFI and TLI estimates. Further, Model 2 showed excellent measurement quality (λ > 0.30; item uniqueness > 0.1 but <0.9) and was thus retained for further analysis.

**Table 3:** Competing confirmatory factor analytical models.

**Model**	**χ^2^**	***df***	**CFI**	**TLI**	**RMSEA**		**SRMR**	**AIC**	**BIC**	**aBIC**	**Meets goodness of fit criteria**	**Meets measurement quality criteria**
Model 0	4970.76	521	0.56	0.53	0.13	[0.125–0.131]	0.15	46629.46	47089.29	46746.47	No	No
Model 1	4104.11	517	0.65	0.62	0.12	[0.112–0.119]	0.13	45770.81	46247.67	45892.15	No	No
Model 2	931.88	491	0.96	0.95	**0.04**	[0.036–0.046]	0.05	42650.58	43238.14	42800.10	Yes	Yes
Model 3	1078.41	508	0.94	0.94	**0.05**	[0.043–0.050]	0.07	42763.11	43278.29	42894.20	Yes	Yes
Model 4	1017.29	497	0.95	0.94	**0.05**	[0.041–0.049]	0.07	42723.98	43286.00	42867.60	Yes	Yes
Model 5	1121.93	512	0.94	0.93	**0.05**	[0.044–0.052]	0.08	42798.62	43296.77	42925.39	Yes	Yes
Model 6	1291.38	512	0.92	0.92	**0.05**	[0.050–0.058]	0.10	42968.07	43466.22	43094.84	Yes	Yes
Model 7	1289.44	512	0.92	0.92	**0.05**	[0.050–0.058]	0.09	42966.14	43464.28	43092.90	Yes	Yes
Model 8	1332.64	515	0.92	0.91	**0.06**	[0.052–0.059]	0.10	43003.34	43488.71	43126.85	Yes	Yes

χ^2^, Chi-square; df, degrees of freedom; TLI, Tucker-Lewis index; CFI, comparative fit index; RMSEA, root mean square error of approximation; SRMR, standardized root mean square residual; AIC, Akaike information criterion; BIC, Bayes information criterion; Bold, non-significant.

### Developing the structural model

**Figure 2:**
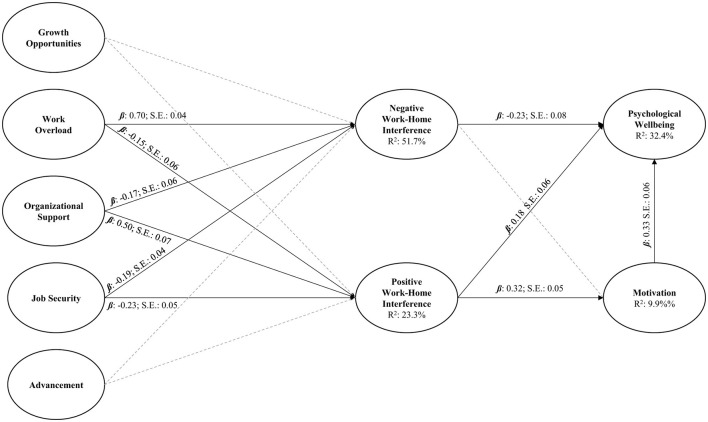
Structural path model.

A structural path model was developed based on the best-fitting measurement Model 2. This model aimed to explore the linear relationships between factors. The structural model is specified in line with the regressive paths shown in Figure 1. Here, work overload, organizational support, growth opportunities, job security, and advancement were specified as exogenous factors, NWHI and PWHI as process factors, and motivation and psychological wellbeing as endogenous factors. This model showed acceptable fit [$\chi_{\left(522\right)}^{2}$ = 11350.08; *df* = 497; CFI = 0.94; TLI = 0.93; RMSEA = 0.05 [0.046–0.053]; *p* > 0.05; SRMR = 0.09; AIC = 42841.78; BIC = 43403.8; aBIC = 42984.79]. The structural model's results are visualized in Figure 2.

The results showed that work overload was positively associated with NWHI (β = 0.70; *SE* = 0.04; *p* < 0.05) and negatively with PWHI (β = −0.15; *SE* = 0.06; *p* < 0.05). Organizational support was negatively associated with NWHI (β = −0.17; *SE* = 0.06; *p* < 0.05) and positively with PWHI (β = 0.50; *SE* = 0.06; *p* < 0.05). Interestingly, job security was negatively related to both NWHI (β = −0.19; *SE* = 0.04; *p* < 0.05) and PWHI (β = −0.23; *SE* = 0.05; *p* < 0.05). Neither growth opportunities nor advancement related to any factor in the model (*p* > 0.05). These exogenous factors cumulatively explained 51.7% of the variance in NWHI and 23.3% of the variance in PWHI.

Similarly, the results showed a negative relationship between NWHI and psychological wellbeing (β = −0.23; *SE* = 0.08; *p* < 0.05), however, no direct relationship to motivation was found. On the other hand, PWHI was directly and positively related to both motivation (β = 0.32; *SE* = 0.05; *p* < 0.05) and psychological wellbeing (β = 0.18; *SE* = 0.06; *p* < 0.05). PWHI explained 9.9% of the variance in motivation. Finally, motivation was also positively associated with psychological wellbeing (β = 0.33; *SE* = 0.06; *p* < 0.05). Cumulatively, these factors explained 32.4% of the overall variance in psychological wellbeing. This structural model was therefore retained for the sequential indirect effects estimation.

In summation, the following hypothesis were accepted:

- **Hypothesis 1 (Accepted)**: Work overload was positively related to NWHI and negatively related to PWHI.
- **Hypothesis 2 (Partially Accepted)**: Organizational support and job security were negatively related to NWHI and organizational support was positively associated with PWHI. However, contrary to expectations, job security was negatively associated with PWHI, and neither growth opportunities nor advancement were significantly associated with either NWHI or PWHI.
- **Hypothesis 3 (Partially Accepted)**: NWHI was negatively related to psychological wellbeing as we hypothesized, however, it did not significantly relate to motivation.
- **Hypothesis 4 (Accepted)**: PWHI positively related to both psychological wellbeing and motivation.
- **Hypothesis 5 (Accepted)**: Motivation positively related to psychological wellbeing.

### Sequential indirect effect estimation: overall and specific effects

The next step was to determine whether NWHI, PWHI, and motivation indirectly affect the relationship between work overload, organizational support, job security, advancement, growth opportunities, and psychological wellbeing. A series of specific and sequential mediation or indirect effect models were estimated based on the structural model. Despite not all hypothesized relationships between exogenous, process, and endogenous factors being significant, Wang and Wang (2020) argued that these should still be included in the overall sequential indirect effects estimation and reported for transparency. The presentation of the results will, therefore only focus on the significant relationships shown in the structural model.

The total indirect effects for the overall sequential mediation paths are presented in Table 4. Table 4 shows that a significant total indirect effect of NWHI, PWHI, and motivation on the relationship between work overload (Estimate = −0.20; SE: 0.07; *p* < 0.05; 95% CI = −0.34 to −0.08), organizational support (Estimate = 0.18; SE: 0.05; *p* < 0.05; 95% CI = 0.10–0.30) and psychological wellbeing is present in the current study. Further, the results showed a significant total indirect effect of motivation on the relationship between PWHI and psychological wellbeing (Estimate = 0.10; SE: 0.03; *p* < 0.05; 95% CI = 0.05–0.17). Given that the confidence interval ranges also did not include zero, there is significant support for the indirect effects. However, the total indirect effect of PWHI, NWHI, and Motivation on the relationship between job security and psychological wellbeing was non-significant (*p* > 0.05), and the confidence interval range included zero. There is, therefore, no support for an overall sequential or serial mediation effect in this relationship.

**Table 4:** Total indirect effects for the overall sequential mediation path.

**Variable**	**Estimate**	***SE***	***t***	***p***	**95% BC CI**	**Meets criteria (overall)**
Overload → psychological wellbeing	−0.20	0.065	−3.13	0.00	[−0.34; −0.08]	Yes
Org. support → psychological wellbeing	0.18	0.052	3.55	0.00	[0.10; 0.30]	Yes
Job security → psychological wellbeing	−0.02	0.033	−0.65	0.52	[−0.09; 0.05]	No
Growth opportunities → psychological wellbeing	−0.03	0.026	−1.14	0.26	[−0.09; 0.02]	No
Advancement → psychological wellbeing	0.00	0.027	−0.04	0.97	[−0.05; 0.05]	No
PWHI → psychological wellbeing	0.10	0.029	3.51	0.00	[0.05; 0.17]	Yes

SE, standard error; BC CI, bias-corrected confidence interval.

When inspecting the specific indirect effects within the path model, there seems to be support for the mediation assumption for several factors (cf. Table 5). First, NWHI indirectly affects the relationship between work overload and psychological wellbeing (Estimate = −0.16; SE: 0.06; *p* < 0.05; 95% CI = −0.30 to −0.04). Similarly, within the sequential model, PWHI and motivation indirectly affect the relationship between work overload and psychological wellbeing (Estimate = −0.02; SE: 0.01; *p* < 0.05; 95% CI = −0.04 to −0.01). Secondly, PWHI indirectly affects the relationship between organizational support and psychological wellbeing (Estimate = 0.09; SE: 0.04; *p* < 0.05; 95% CI = 0.03–0.18). Within the sequential model, both PWHI and motivation indirectly affect the relationship between organizational support and psychological wellbeing (Estimate = 0.05; SE: 0.02; *p* < 0.05; 95% CI = 0.02–0.10). Third, motivation indirectly affects the relationship between PWHI and psychological wellbeing (Estimate = 0.10; SE: 0.03; *p* < 0.05; 95% CI = 0.05–0.17).

**Table 5:** Indirect effects for specific mediation paths.

**Variable**	**Estimate**	***SE***	***t***	***p***	**95% BC CI**		**Meets criteria (specific)**
					**LCI**	**UCI**	
Overload → NWHI → PWB	−0.16	0.06	2.58	0.01	−0.30	−0.04	Yes
Overload → PWHI → PWB	−0.03	0.02	−1.76	0.08	−0.07	−0.01	No
Overload → NWHI → motivation → PWB	0.00	0.01	0.08	0.94	−0.03	0.03	No
Overload → PWHI → motivation → PWB	−0.02	0.01	−1.97	0.05	−0.04	−0.01	Yes
Org. support → NWHI → PWB	0.04	0.02	1.82	0.07	0.01	0.10	No
Org. support → PWHI → PWB	0.09	0.04	2.38	0.02	0.03	0.18	Yes
Org. support → NWHI → motivation → PWB	0.00	0.00	−0.08	0.94	−0.01	0.01	No
Org. support → PWHI → motivation → PWB	0.05	0.02	2.58	0.01	0.02	0.10	Yes
Job security → NWHI → PWB	0.04	0.02	2.16	0.03	0.01	0.10	Yes
Job security → PWHI → PWB	−0.04	0.02	−2.23	0.03	−0.09	−0.01	Yes
Job security → NWHI → motivation → PWB	0.00	0.00	−0.08	0.94	−0.01	0.01	No
Job security → PWHI → motivation → PWB	−0.02	0.01	−2.65	0.01	−0.05	−0.01	Yes
Growth opportunities → NWHI → PWB	−0.02	0.02	−1.42	0.16	−0.07	0.00	No
Growth opportunities → PWHI → PWB	−0.01	0.01	−0.38	0.70	−0.04	0.02	No
Growth opportunities → NWHI → motivation → PWB	0.00	0.00	0.07	0.94	0.00	0.01	No
Growth opportunities → PWHI → motivation → PWB	0.00	0.01	−0.39	0.69	−0.02	0.01	No
Advancement → NWHI → PWB	0.02	0.02	1.21	0.23	0.00	0.06	No
Advancement → PWHI → PWB	−0.01	0.01	−0.94	0.35	−0.05	0.01	No
Advancement → NWHI → motivation → PWB	0.00	0.00	−0.07	0.95	−0.01	0.00	No
Advancement → PWHI → motivation → PWB	−0.01	0.01	−1.02	0.31	−0.02	0.01	No
PWHI → motivation → PWB	0.10	0.03	3.51	0.00	0.05	0.17	Yes

SE, standard error; p, obtained significance value; LCI, lower confidence interval; UCI, upper confidence interval.

Finally, although support was found for an overall indirect effect between job security and psychological wellbeing, the results showed support for only three of the four specific paths (cf. Table 6). The results showed that NWHI (Estimate = 0.04; SE: 0.02; *p* < 0.05; 95% CI = 0.01–0.10) and PWHI (Estimate = −0.04; SE: 0.02; *p* < 0.05; 95% CI = −0.09 to −0.01) indirectly affect the relationship between job security and psychological wellbeing. The results also show that PWHI and motivation indirectly affect the relationship between job security and psychological wellbeing (Estimate = −0.02; SE: 0.01; *p* < 0.05; 95% CI = −0.05 to −0.01).

**Table 6:** Regression results for the moderation effect—NWHI.

**Variable**	**Estimate**	***SE***	***t***	***p***	**95% BC CI**		**Meets criteria (specific)**
					**LCI**	**UCI**	
Constant	0.00	0.02	−0.08	0.94	−0.043	0.040	No
Overload	0.84	0.03	26.57	0.00	0.775	0.899	
Strengths use	−0.26	0.03	−8.18	0.00	−0.323	−0.198	
Overload × strengths use	0.02	0.04	0.51	0.61	−0.059	0.101	
Constant	0.00	0.04	−0.10	0.92	−0.076	0.068	No
Organizational support	0.03	0.06	0.50	0.62	−0.089	0.149	
Strengths use	−0.14	0.06	−2.13	0.03	−0.265	−0.011	
Organizational support × strengths use	0.01	0.06	0.21	0.84	−0.100	0.123	
Constant	−0.01	0.03	−0.42	0.67	−0.076	0.049	Yes
Job security	−0.15	0.03	−5.27	0.00	−0.206	−0.094	
Strengths use	−0.05	0.05	−1.06	0.29	−0.145	0.043	
Job security × strengths use	0.08	0.04	2.18	0.03	0.008	0.155	
Constant	0.00	0.02	0.08	0.94	−0.042	0.045	No
Overload	0.85	0.03	25.79	0.00	0.784	0.913	
Job crafting	−0.29	0.05	−6.52	0.00	−0.383	−0.206	
Overload × job crafting	−0.02	0.06	−0.33	0.74	−0.142	0.101	
Constant	0.01	0.04	0.36	0.72	−0.061	0.088	No
Organizational support	−0.14	0.07	−2.01	0.04	−0.273	−0.003	
Job crafting	0.14	0.10	1.43	0.15	−0.053	0.334	
Organizational support × job crafting	−0.05	0.08	−0.69	0.49	−0.205	0.099	
Constant	0.00	0.03	−0.01	0.99	−0.062	0.061	Yes
Job security	−0.16	0.03	−5.92	0.00	−0.218	−0.110	
Job crafting	0.02	0.06	0.37	0.71	−0.101	0.148	
Job security × job crafting	0.15	0.05	2.96	0.00	0.051	0.254	
Constant	0.00	0.02	−0.11	0.91	−0.046	0.041	No
Overload	0.80	0.03	24.19	0.00	0.734	0.864	
Positive home-work interference	0.03	0.02	1.24	0.22	−0.016	0.072	
Overload × positive home-work interference	−0.05	0.03	−1.60	0.11	−0.107	0.011	
Constant	0.01	0.03	0.21	0.83	−0.057	0.071	No
Organizational support	−0.06	0.05	−1.32	0.19	−0.154	0.030	
Positive home-work interference	0.00	0.03	−0.12	0.91	−0.068	0.061	
Organizational support × positive home-work interference	−0.06	0.05	−1.38	0.17	−0.151	0.027	
Constant	0.02	0.03	0.50	0.62	−0.048	0.081	No
Job security	−0.20	0.03	−6.56	0.00	−0.255	−0.137	
Positive home-work interference	−0.09	0.03	−2.72	0.01	−0.157	−0.025	
Job security × negative home-work interference	0.04	0.03	1.52	0.13	−0.013	0.100	
Constant	0.00	0.02	0.15	0.88	−0.030	0.035	No
Overload	0.71	0.02	28.27	0.00	0.656	0.754	
Negative home-work interference	0.68	0.03	20.64	0.00	0.614	0.744	
Overload × negative home-work interference	−0.04	0.05	−0.88	0.38	−0.141	0.054	
Constant	0.00	0.03	0.01	0.99	−0.053	0.053	No
Organizational support	0.09	0.04	2.22	0.03	0.010	0.161	
Negative home-work interference	0.86	0.05	16.07	0.00	0.757	0.968	
Organizational support × negative home-work interference	0.00	0.07	0.05	0.96	−0.135	0.142	
Constant	0.01	0.03	0.42	0.67	−0.045	0.070	No
Job security	0.03	0.03	1.12	0.26	−0.023	0.084	
Negative home-work interference	0.89	0.06	14.26	0.00	0.768	1.013	
Job security × negative home-work interference	0.04	0.04	1.00	0.32	−0.042	0.129	

SE, standard error; p, obtained significance value; LCI, lower confidence interval; UCI, upper confidence interval.

In summation, the following hypothesis were accepted:

- **Hypothesis 6 (Partially Accepted)**: Positive and negative work-home interference, along with motivation, sequentially mediated the relationships between job characteristics (work overload, organizational support) and psychological wellbeing. However, this sequential mediation was not supported for growth opportunities and advancement, nor could be fully supported for job security.

### Interaction effect estimation: strength use, job crafting, and home-work interaction

The final objective of this study was to investigate whether strengths use, job crafting, and home-work interaction (PHWI and NHWI) could moderate the relationship between job characteristics (work overload, organizational support, job security, growth opportunities, and advancement) and work-home interaction (PWHI and NWHI). The factor scores of the best-fitting measurement model were used as input into Hayes' (2017) hierarchical regression procedure to estimate interaction effects.

Table 6 provides a summary of the regression results for the interaction effects between the moderators (strengths use, job crafting, PHWI, NHWI) and the factors (work overload, organizational support, job security) that showed to be significantly related to NWHI in the structural model. The results show that only strengths use and job crafting moderated the relationship between job security and NWHI. No other interaction effect significantly impacted the relationship between job characteristics and NWHI.

**Figure 3:**
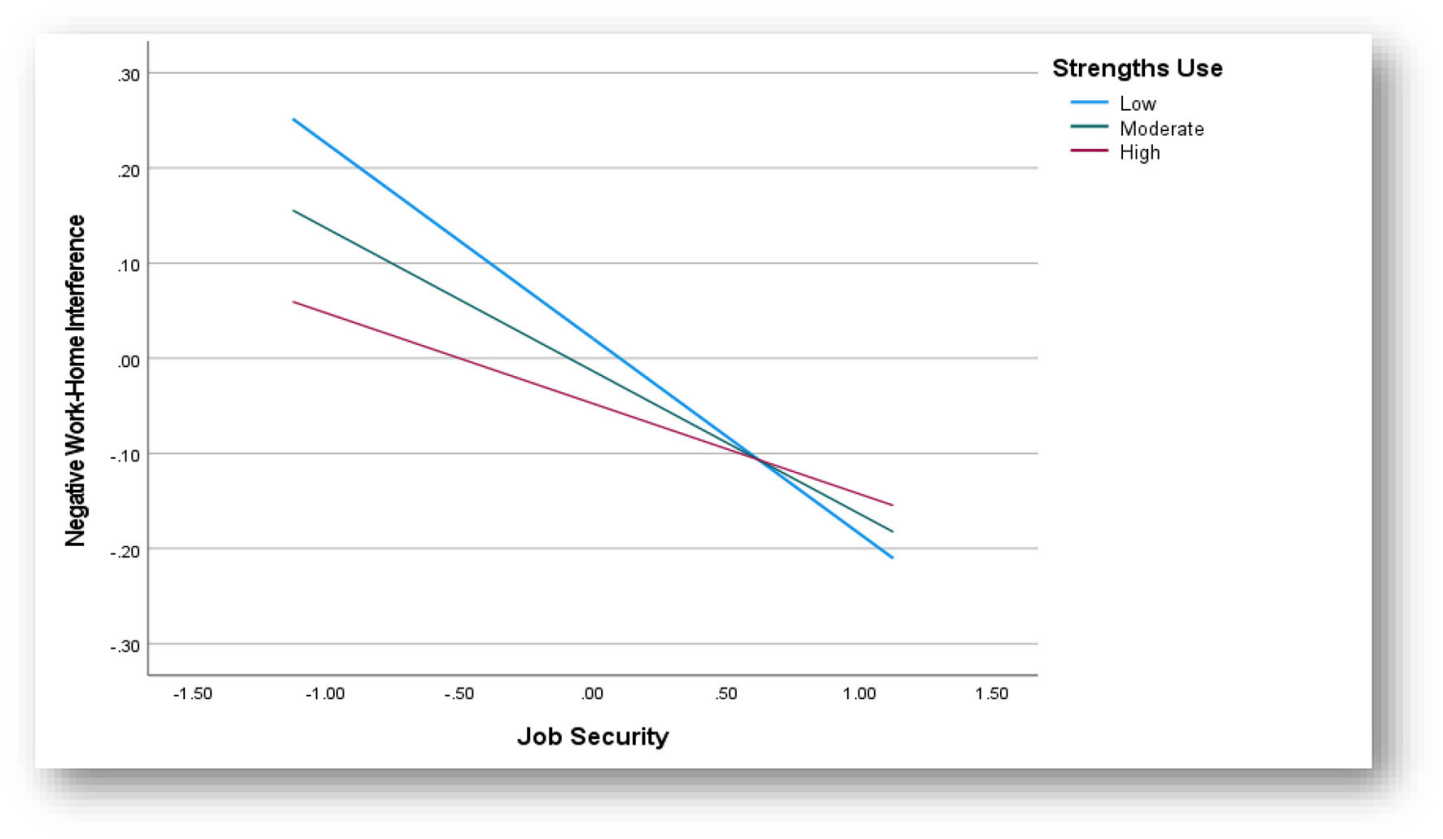
Interaction effect—Strength use.

In the first instance, both job security and strengths use accounted for a significant proportion of the variance in NWHI [*F*_(3, 518)_= 13.69; *p* < 0.05; *R*^2^ = 7.4%]. When the interaction term [job security × strengths use; *F*_(1, 518)_= 4.75; β = 0.08; *SE* = 0.04; *p* = 0.03; *t* = 2.18; Δ*R*^2^ = 0.85%] was added to the model, an additional 0.85% of the variance was explained. Further, the 95% CI range of the interaction term did not include zero (LCI = 0.01; UCI = 0.16). A follow-up simple slopes analysis (at ±1 SD of the moderator) demonstrated that the negative relationship between job security and NWHI was weakened when individuals showed increasing levels of strengths-use. Specifically, when employees show high levels of strength use, the relationship seems to be weaker for those who reported high (β = −0.10, SE = 0.04; *p* < 0.05; 95% CI = −0.17 to −0.02) vs. average (β = −0.15, SE = 0.03; *p* < 0.05; 95% CI = −0.21 to −0.08) and low levels of strengths use (β = −0.21, SE = 0.04; *p* < 0.05; 95% CI = −0.28 to −0.13). There is thus support that the relationship between job security and NWHI is moderated by strengths use (cf. Figure 3).

**Figure 4:**
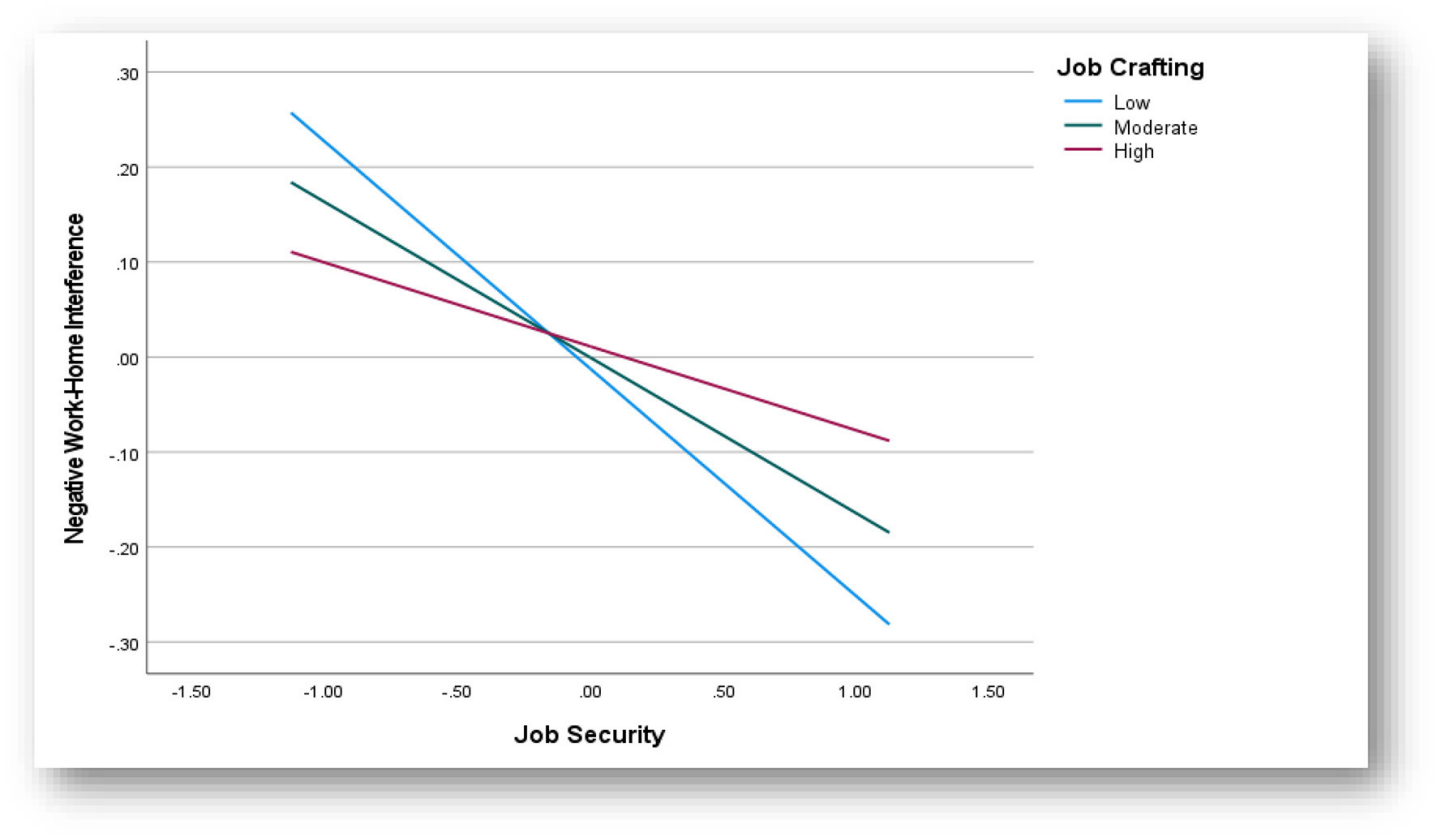
Interaction effect—Job crafting.

Similarly, both job security and job crafting accounted for a significant proportion of the variance in NWHI [*F*_(3, 518)_= 14.47; *p* < 0.05; *R*^2^ = 7.7%]. When the interaction term [job security × job crafting; *F*_(1, 518)_= 8.76; β = 0.15; *SE* = 0.05; *p* = 0.00; *t* = 2.96; Δ*R*^2^ = 1.6%] was added to the model, an additional 1.6% of the variance was explained. Further, the 95% CI range of the interaction term did not include zero (LCI = 0.05; UCI = 0.25). A simple slopes analysis (at ±1 SD of the moderator) further showed that the negative relationship between job security and NWHI was weakened when individuals showed increasing levels of job crafting. The results showed that when employees reported high levels of job crafting, the relationship was weaker (β = −0.09, SE = 0.04; *p* < 0.05; 95% CI = −0.16 to −0.02) than when they reported average (β = −0.16, SE = 0.03; *p* < 0.05; 95% CI = −0.22 to −0.11) or low levels (β = −0.24, SE = 0.04; *p* < 0.05; 95% CI = −0.31 to −0.17). There is thus support that the relationship between job security and NWHI is moderated by job crafting (cf. Figure 4).

Table 7 summarizes the regression results for the moderation effects between the factors that were significantly related to PWHI. None of the moderating factors (job crafting, strengths use, PHWI, NWHI) statistically significantly affected the relationship between job characteristics (work overload, organizational support, job security) and PWHI. There is, therefore, no support for that job crafting, strengths use, PHWI, and NWHI buffer or enhance the relationship between job characteristics and PWHI.

**Table 7:** Regression results for the moderation effect—PWHI.

**Variable**	**Estimate**	***SE***	***t***	***p***	**95% BC CI**		**Meets criteria (specific)**
					**LCI**	**UCI**	
Constant	0.00	0.01	0.02	0.99	−0.022	0.022	No
Overload	−0.05	0.02	−3.08	0.00	−0.083	−0.018	
Strengths use	0.17	0.02	10.23	0.00	0.137	0.202	
Overload × strengths use	0.00	0.02	−0.10	0.92	−0.044	0.040	
Constant	0.00	0.01	−0.23	0.82	−0.027	0.021	No
Organizational support	0.09	0.02	4.17	0.00	0.045	0.125	
Strengths use	0.11	0.02	4.88	0.00	0.063	0.149	
Organizational support × strengths use	0.01	0.02	0.48	0.63	−0.028	0.047	
Constant	0.00	0.01	0.02	0.99	−0.021	0.021	No
Job security	−0.07	0.01	−7.85	0.00	−0.093	−0.056	
Strengths use	0.19	0.02	11.85	0.00	0.157	0.220	
Job security × strengths use	0.00	0.01	−0.07	0.94	−0.025	0.024	
Constant	0.00	0.01	0.22	0.82	−0.017	0.022	No
Overload	−0.09	0.01	−5.99	0.00	−0.116	−0.059	
Job crafting	0.34	0.02	17.21	0.00	0.305	0.384	
Overload × job crafting	−0.03	0.03	−0.95	0.35	−0.080	0.028	
Constant	0.00	0.01	−0.15	0.88	−0.024	0.021	No
Organizational support	−0.03	0.02	−1.59	0.11	−0.074	0.008	
Job crafting	0.35	0.03	11.77	0.00	0.293	0.411	
Organizational support × job crafting	0.01	0.02	0.28	0.78	−0.040	0.053	
Constant	0.00	0.01	0.00	1.00	−0.019	0.019	No
Job security	−0.05	0.01	−5.94	0.00	−0.067	−0.034	
Job crafting	0.32	0.02	16.33	0.00	0.277	0.353	
Job security × job crafting	−0.01	0.02	−0.51	0.61	−0.039	0.023	
Constant	0.00	0.01	−0.03	0.98	−0.013	0.013	No
Overload	0.00	0.01	0.47	0.64	−0.015	0.024	
Positive home-work interference	0.23	0.01	34.56	0.00	0.216	0.242	
Overload × positive home-work interference	0.00	0.01	−0.43	0.66	−0.021	0.014	
Constant	0.00	0.01	0.04	0.97	−0.011	0.012	No
Organizational support	0.10	0.01	11.92	0.00	0.084	0.117	
Positive home-work interference	0.22	0.01	36.79	0.00	0.206	0.229	
Organizational support × positive home-work interference	0.00	0.01	−0.23	0.82	−0.018	0.014	
Constant	0.00	0.01	−0.30	0.76	−0.016	0.011	No
Job security	0.02	0.01	3.53	0.00	0.010	0.034	
Positive home-work interference	0.24	0.01	33.95	0.00	0.224	0.252	
Job security × negative home-work interference	−0.01	0.01	−0.92	0.36	−0.017	0.006	
Constant	0.00	0.01	0.09	0.93	−0.023	0.025	No
Overload	−0.03	0.02	−1.41	0.16	−0.061	0.010	
Negative home-work interference	0.02	0.02	0.94	0.35	−0.024	0.069	
Overload × negative home-work interference	−0.02	0.04	−0.52	0.60	−0.089	0.052	
Constant	0.00	0.01	−0.12	0.90	−0.023	0.021	No
Organizational support	0.16	0.02	10.05	0.00	0.130	0.193	
Negative home-work interference	0.07	0.02	2.92	0.00	0.021	0.109	
Organizational support × negative home-work interference	−0.02	0.03	−0.56	0.58	−0.074	0.041	
Constant	0.00	0.01	0.14	0.89	−0.024	0.027	No
Job security	−0.06	0.01	−5.13	0.00	−0.086	−0.038	
Negative home-work interference	−0.05	0.03	−1.75	0.08	−0.103	0.006	
Job security × negative home-work interference	0.01	0.02	0.33	0.74	−0.031	0.044	

SE, standard error; p, obtained significance value; LCI, lower confidence interval; UCI, upper confidence interval.

In summation, the following hypothesis were accepted:

- **Hypothesis 7 (Partially Accepted)**: Strengths use and job crafting only moderated the relationship between job security and NWHI. Specifically, higher levels of strengths use and job crafting weakened the negative relationship between job security and NWHI. However, strengths use and job crafting did not moderate the relationship between other job characteristics (work overload, organizational support, growth opportunities, advancement) and work-home interference.
- **Hypothesis 7 (Rejected)**: Neither positive nor negative home-work interference significantly moderated the relationships between job characteristics and work-home interference.

## Discussion

This study investigated how job characteristics, work-home interference, and motivation influenced psychological wellbeing during the COVID-19 lockdown, as well as the moderating effects of strengths use, job crafting, and home-work interference. The findings provide important insights into the complex interplay of these factors during a crisis, highlighting both direct and indirect relationships that contribute to employee wellbeing. The results reveal that work overload, organizational support, and job security are significant determinants of work-home interference (both positive and negative), while growth opportunities and advancement played a limited role. Additionally, positive and negative work-home interference were directly associated with psychological wellbeing, while motivation was linked to wellbeing through positive spillover effects. However, the moderating effects of strengths use and job crafting were only evident in the relationship between job security and negative work-home interference, suggesting their limited role during the pandemic. Overall, the study suggests that neither job crafting nor strength use can be used as effective strategies for managing the work-home interface issues that job characteristics may cause during times of crisis. Further, the outcomes of home regulatory practices like the positive/negative home-work spill may not be helpful in explaining how job characteristics affect the work-home relationship. Organizations may therefore need to focus on addressing workload, organizational support and job security directly, rather than relying solely on individual-level interventions to improve work-life balance and wellbeing of employees.

### Job characteristics and work-home interaction

The results indicate that job demands and resources played a pivotal role in shaping work-home dynamics during the lockdown period.

*Work overload* emerged as a key driver of NWHI, while simultaneously reducing PWHI. This finding aligns with the JDR framework's health impairment process, which posits that excessive demands deplete employees' mental and physical resources, making it challenging to maintain a balance between work and personal responsibilities (Bakker et al., 2023). The unique context of remote work during the pandemic likely exacerbated these effects, as blurred boundaries between home and work settings increased role conflicts and time pressures (Schieman et al., 2021).

*Organizational support* was found to be negatively related to negative- and positively to positive work-home interference during the COVID-19 lockdowns. This implies that the more support an employee receives from their organization, the less likely they are to experience negative- and the more likely they are to experience positive interference between their work and personal life. There are several potential reasons for these relationships. Employees who feel supported by their organizations may be more likely to have access to the physical- and social resources required to manage their work-related tasks more effectively (De Klerk et al., 2021). They may have had more flexibility in work arrangements and more support from colleagues/managers. This helps effectively navigate the challenges brought on by the pandemic by allowing more flexibility in managing work-related responsibilities, making it easier to attend to home-related tasks (Yang et al., 2023). In contrast, employees who may have experienced low support from their organizations may not have had access to the resources required to perform, which may have been exasperated by the increased job demands such as high workloads, working longer hours, and little understanding of managers as to the personal challenges employees were facing (De Klerk et al., 2021).

Further, a negative relationship between *job security* and both negative- and positive work-home interference was found in this study. In times of economic uncertainty, employees are more likely to report low levels of security about their ability to maintain gainful employment, which ultimately negatively affects their personal lives. These reports of low job security create a sense of anxiety and uncertainty which interferes with employees' ability to focus on non-work-related activities (Bakker et al., 2023). This may lead to more negative work-home interference, as meeting personal performance targets become an overarching life priority as it's used as a means to maintain gainful employment. This over-prioritization of work over one's personal life results in poor social relationships, increases the number of hours spent at work or engaged in work-related activities, reduces recovery time and decreases personal autonomy (Demerouti and Bakker, 2022). Further the negative relationship between job security and positive work-home interference implies that when employees feel that their jobs are secure, they are less likely to experience a positive spill-over of resources into their private lives. This may be due to the increased levels of personal responsibility taken over work-related tasks of those with job security during the pandemic (Bakker et al., 2023). Individuals with job security were more likely to take ownership of work-related tasks and use such as a means to justify their employment status within the company.

In contrast to initial expectations, neither *growth opportunities* nor *advancement* was shown to relate to negative- or positive work-home interference. There are several potential reasons for such. Growth opportunities are traditionally seen as a means to facilitate professional development and advancement, but both increased job demands and limited access to these opportunities during the lockdowns may not necessarily translate to reduced or increased work-home interference (Bakker et al., 2023). Further, challenges brought on by the pandemic (e.g., learning new technologies and communication tools, increased social isolation etc.) may have overshadowed the perceived benefits of investing in professional growth activities, which limited the potential effect it may have had on work-home interference. Similarly, the COVID-19 pandemic may have halted advancement opportunities as the focus was on changing internal systems, processes, and procedures to maintain the status quo and keep the business running. Further, it may also be possible that the increased flexibility in the execution of work-related tasks and autonomy may have been more valued, thus shifting the focus from career progression to maintaining a better work-life balance (Yang et al., 2023). Further, other factors, such as organizational support and job security had a stronger impact on work-home interference than growth opportunities, given the uncertainties and stressors associated with the pandemic.

### Work-home interaction, motivation, and psychological wellbeing

Our results showed that positive- and negative work-home interaction and motivation were important conditions affecting employees' psychological wellbeing. Similarly, positive work-home interference showed to be significantly related to motivation.

*Negative work-home interaction* was found to relate negatively to psychological wellbeing. When work demands and responsibilities intrude into one's personal life, it leads to increased experiences of stress and strain, which ultimately negatively affects their psychological wellbeing. This may be due to the difficulty individuals experience in detaching themselves from work-related issues, resulting in reduced recovery time and increased psychological strain (Bakker et al., 2023). Sudden changes in work arrangements, approaches and styles and prolonged exposure to work-related stressors increase reports of emotional, physical, and psychological exhaustion (Demerouti and Bakker, 2022). This may have enduring effects on employee's overall psychological wellbeing and mental health (Bakker et al., 2023).

Similarly, our results showed that *positive work-home interference* related positively to both psychological wellbeing and motivation. When employees perceive their work experiences as enriching their personal lives, it fosters a sense of personal fulfillment, ultimately increasing motivation and overall wellbeing (Bakker et al., 2023). Positive work-home interference may allow individuals to draw on positive emotions, and personal enriching experiences at work as a means to motivate them to perform at work and to function better in their daily lives. Further, increased positive experiences at work may increase employees' self-efficacy and enhance their belief in their own abilities, which in turn helps to better manage work-related challenges. This in turn may enhance their motivation and psychological wellbeing (Demerouti and Bakker, 2022).

Finally, *motivation* was also positively associated with psychological wellbeing. When motivated, individuals can mobilize and allocate their efforts and attention to achieving personal and professional goals that they perceive to be personally meaningful. Motivated employees are more likely to harbor a positive outlook on life and report a greater sense of accomplishment and higher levels of work-related self-efficacy, which helps them deal effectively with work-related setbacks and challenges. This in turn increases experiences of overall psychological wellbeing (De Klerk et al., 2021).

### How work-home interference and motivation indirectly affect the relationship between job characteristics and psychological wellbeing

The study also found some support for the extent to which work-home interference and motivation indirectly affect the relationship between job characteristics and psychological wellbeing. Sequential mediation effects were found for the overall pathways between work overload, organizational support, and psychological wellbeing through negative/positive work-home interference and motivation. Similarly, motivation indirectly affected the relationship between positive work-home interference and psychological wellbeing. When inspecting the specific indirect paths of the non-significant overall effects models, the results showed that negative work-home interference indirectly affected the relationship between job security and psychological wellbeing and that positive work-home interference and motivation affected the relationship between job security and psychological wellbeing.

The first finding suggests that negative work-home interference indirectly affected the relationship between work overload and psychological wellbeing. When individuals experience high levels of work overload, they may struggle to effectively balance their work and personal life, which creates conflicts and challenges in fulfilling personal responsibilities or life tasks. This negative spill-over between work and home domains creates additional emotional strain, further impacting their psychological wellbeing (Demerouti and Bakker, 2022). Similarly, positive work-home interference and motivation were found to have a cumulative indirect effect on the relationship between work overload and psychological wellbeing. High work overload reduces the likelihood of experiencing positive work-home spill-over, decreasing motivation and subsequently affecting psychological wellbeing.

The second finding highlights that work-home interference and motivation indirectly affect the relationship between organizational support and psychological wellbeing. Although the overall sequential mediation model was significant, only the sequential path through positive work-home interference and motivation was significant. This implies that when individuals feel that the organization values their contributions and provides the necessary emotional and instrumental support, they are more likely to experience positive emotional experiences at work, spilling over into personal life domains (Bakker et al., 2023). When these positive experiences at work spill-over into one's personal life, individuals are more likely to be motivated at work, increasing their psychological wellbeing.

The third finding suggests that motivation indirectly affects the relationship between positive work-home interference and psychological wellbeing. When employees perceive a positive spill-over from aspects of work into their private lives, they are more likely to feel motivated, which in turn increases their psychological wellbeing. This finding aligns with the basic tenets of self-determination theory, which posits that when work-related experiences and accomplishments spill-over into their personal lives, it can fulfill one's need for competence and relatedness (Ryan and Deci, 2001). This positive spill-over leads to feelings of accomplishment, satisfaction, and connectedness, which may contribute to overall motivation. Motivated employees are driven to pursue meaningful goals and invest effort in accomplishing work tasks which helps maintain positive affective states which are vital components of psychological wellbeing.

Finally, the overall sequential mediation effect between job security and psychological wellbeing was not significant, indicating that negative and positive work-home interference and motivation do not collectively mediate the relationship. However, when inspecting the specific effects, the results demonstrate that the direct relationship between job security and psychological wellbeing is mediated by negative work-home interference and that positive work-home interference and motivation collectively also indirectly affect this relationship. This implies that when there is certainty about one's position within the company, employees are less likely to experience a negative spill-over of work into their private lives, which in turn increases their psychological wellbeing. Similarly, when there is greater certainty about the ability to maintain gainful employment, people are less likely to experience a positive spill-over effect from work to home, which affects their motivation and psychological wellbeing (as explained in the previous section).

### The role of job crafting, strengths use, and home-work interaction

The final objective of this study was to determine whether personal resources (such as strengths use), proactive individual regulatory practices (such as job crafting) and how the outcomes of proactive- and destructive home regulatory strategies (positive- and negative home-work interference) can help employees effectively navigate the challenges of remote work during times of crisis. The findings only supported the effect of strengths use and job crafting as moderators on the relationship between job security and negative work-home interaction. No other moderating effect of strengths use, job crafting and positive/negative home-work interaction on the relationship between job characteristics and work-home spill-over could be established.

The main finding suggests that when individuals are able to use their strengths at work and when they are able to craft their jobs, it strengthens the effect job security has on negative work-home interference. When individuals are able to effectively use their strengths at work, they may be better equipped to cope with the negative effects low job security has on work-home interference. By recognizing and leveraging their strengths, individuals are better equipped to optimize their available resources, thereby mitigating the negative spill-over effects of work into their private lives during the challenges of remote working.

Moreover, the findings demonstrate that job crafting acts as another significant moderator in the relationship between job security and negative work-home interference. When individuals proactively modify their job tasks, work relationships, and perceptions of the purpose of work to better suit their needs, it diminishes the influence of low job security on creating negative work-home spill-over (Van Zyl et al., 2023). This suggests that individuals who can craft their jobs to better suit their personal needs/preferences establish a better fit between their work and home domains, thus ameliorating the adverse effects of low job security on the spillage of work-related stressors into their home lives. These findings highlight the importance of individual agency and the ability to shape one's work environment in the context of forced remote work. Empowering employees to leverage their strengths and engage in job crafting practices can serve as effective strategies to mitigate the negative consequences of low job security on work-home interference, ultimately promoting employees' balance between work and personal life and wellbeing during times of crisis.

In contrast to initial expectations, our findings did not find support for the moderating role of strengths use, job crafting, or positive/negative home-work interference on the relationship between job characteristics (work overload, organizational support, advancement, growth opportunities) and positive/negative work-home spill-over during the COVID-19 lockdowns. These findings suggest that during the unprecedented period of forced remote work, neither personal resources nor individual regulatory practices and the outcomes of home regulatory factors significantly impact how job characteristics influence the spill-over between work and home domains.

One explanation for these non-significant findings could be the unique circumstances and challenges posed by the COVID-19 lockdowns. The unique stressors and challenges brought on by the pandemic, such as health concerns, increased caregiving responsibilities, and limited social interactions, may have overshadowed the impact of personal resources, individual regulatory practices and home-work interference had on the work-home spill-over. The sudden shift to remote work and the accompanying disruptions to work routines and boundaries may have overshadowed the potential moderating effects of strengths use, job crafting, and positive/negative home-work interference as individuals struggled to adapt to new working conditions and navigate the uncertainties brought about by the pandemic. Moreover, the increased blurring of boundaries between work and home due to remote work might have diminished the positive/negative home-work interference's influence on the relationship between job characteristics and work-home spill-over.

### Cracks in the JDR: theoretical and practical implications

#### Theoretical implications

Our findings necessitate a critical re-examination of the Job Demands-Resources (JD-R) framework, particularly in contexts characterized by severe unpredictability, structural disruptions, and crisis conditions like the COVID-19 pandemic. Although the JD-R model has traditionally provided robust insights into the interplay between job demands, resources, and employee wellbeing (Demerouti and Bakker, 2022; Geurts et al., 2005), our results suggest that its explanatory power may be substantially diminished when work environments are subject to extreme and unpredictable pressures. Schaufeli and Taris (2014) have previously highlighted the model's conceptual ambiguity regarding what constitutes demands vs. resources, and its impact on wellbeing, but our study extends this critique by demonstrating that these categorizations may become even more fluid during crises.

First, our findings compel us to question the universality of the JD-R model, as the structural demands of a crisis appear to override what we could consider the typical wellbeing benefits that's usually associated with certain job resources. Given that our results only partially supported assumptions by Demerouti and Bakker (2022) about how the relationships between job demands, resources, work-life balance and wellbeing would manifest in times of crisis, we have to re-consider the importance of job resources (like growth opportunities and advancement) when trying to explain how work life spills-over into one's personal life and how this affects motivation and wellbeing during the COVID-19 pandemic. The pandemic seems to have altered the perceptive importance of growth opportunities and advancement as the focus may have shifted away from professional growth initiatives and career advancement to more active management of job demands and creating clearer work and life boundaries (Yang et al., 2023). The findings imply that the structural components of work, which are usually taken for granted under more stable conditions, may require reconfiguration to accommodate the shifting priorities of employees when they are facing acute stressors brought on in times of uncertainty, change, and crisis.

Moreover, our findings regarding growth opportunities and advancement challenge the JD-R model's presumption that these resources invariably contribute to motivational processes. Crawford et al.'s (2010) meta-analysis previously suggested that certain demands could be appraised as challenges rather than hindrances, contributing to engagement rather than burnout. Our study inverts this insight, suggesting that certain resources may lose their motivational potential during crises, becoming irrelevant or even counterproductive to wellbeing outcomes. This further problematizes the JD-R model's categorization of job characteristics as either demands or resources (Taris and Schaufeli, 2016), suggesting instead that their functional impact may be contextually dependent and temporally unstable.

Further, job security is typically heralded as a stabilizing resource within the JDR framework as it is argued to be essential for ensuring better work-life balance and improving wellbeing (Demerouti and Bakker, 2022). Paradoxically, our data indicate that job security was associated with both increased negative work-home spillover and a reduction in positive spillover. One possible interpretation is that employees with secure positions may have experienced an intensified sense of duty by taking on additional tasks or perceived an increased pressure to perform to justify their positions which further blurred the boundaries between work and personal life (Yang et al., 2023). Hobfoll's (1989) Conservation of Resources theory may offer a complementary explanation: during periods of severe resource threat, individuals with more resources to lose (i.e., secure employment) may experience heightened anxiety about potential resource depletion, triggering compensatory behaviors that inadvertently exacerbate work-home conflicts. Thus, while job security might traditionally be seen as a buffer against work stress, in crisis contexts it may inadvertently contribute to overcommitment and further exacerbate work-home conflicts.

An even more fundamental critique emerging from our findings concerns the JD-R model's implicit assumption that individual agency and resources can effectively counterbalance structural demands (Bakker and Demerouti, 2018). Our results question Demerouti and Bakker's (2022) declaration that as to the universal, unequivocal importance of personal resources, individual regulatory strategies and the outcomes of proactive and reactive home regulatory factors for improving individuals' wellbeing during the times of crisis. Where Demerouti and Bakker (2022) indicated that strengths use, job crafting and the home-work spill over may help individuals cope with the challenges of remote working brought on by the pandemic, our results showed that addressing demands and resources directly seemed to be more suitable. Our findings challenge the prevalent narrative within positive organizational scholarship that personal resources and proactive behaviors can compensate for adverse working conditions. In the face of systemic and structural issues, especially in times of crisis, expecting employees to rely solely on their own individual regulatory strategies is not only inadequate to help offset the pressures that unexpected crisis brings but is also an *unfair and unjust transfer* of responsibility from the organization to the individual to cope with these issues. It is, however, important to note that we did not measure nor control for the available opportunities to use strengths or the effectiveness of one's ability to craft one's job. The forced remote work may have limited access to opportunities to engage in these behaviors, thus limiting their potential impact on managing the effects job characteristics had on work-home interference.

Additionally, Vanbelle et al. (2017) have argued, the efficacy of positive psychological interventions may be bounded by structural constraints. Our findings extend this critique by demonstrating that during systemic crises, the compensatory capacity of individual resources may be severely constrained. This resonates with Lomas et al.'s (2019) systematic review of workplace mindfulness interventions, which found that while individually focused interventions might improve subjective wellbeing, they often fail to address the structural determinants of workplace stress. Similarly, Van den Heuvel et al. (2015) questioned whether job crafting could effectively mitigate systemic organizational challenges, particularly when employees' autonomy is restricted by crisis-induced constraints. These results also align with broader sociological critiques of positive psychology's tendency toward “responsibilization” of wellbeing (Cabanas and Illouz, 2019), wherein structural problems are reframed as individual challenges amenable to personal solutions. Our study provides empirical support for Bal and Dóci's (2018) contention that the JDR framework, despite its contributions, risks inadvertently legitimizing workplace intensification by suggesting that its negative effects can be neutralized through individual resources and strategies. This critique is particularly salient given our findings that even traditionally effective individual regulatory strategies showed minimal buffering effects during the pandemic.

This perspective aligns with critical scholarship questioning the assumption that individual focused strategies and coping should be the primary response to systemic workplace pressures (Van Zyl et al., 2024a,b). An overemphasis on individual coping strategies may divert attention from necessary organizational changes, potentially leading “resilience washing” (i.e., the practice of promoting individual adaptability as a substitute for addressing problematic work structures). Our results provide some empirical support of other studies questioning the nature and role of personal resources and individual regulatory strategies within the JDR framework (e.g., Gregory et al., 2010; Schaufeli, 2017; Teuber et al., 2021; Van Zyl et al., 2021a). Specifically, we provide some support for arguments suggesting that during the pandemic, structural job characteristics exerted their effects on wellbeing largely independent of individual regulatory strategies. Taken together, there is thus a critical need to further explore the function of these personal factors, and their underlying mechanisms within the health impairment and motivational processes of the JDR framework.

#### Practical implications

From a practical standpoint, our findings underscore the imperative for organizations to reorient their approach to their intervention strategies. Instead of relying predominantly on improving employees' individual coping strategies, an approach that Nielsen and Miraglia (2017) describe as potentially “blaming the victim,” there is a critical need for systemic changes during times of crisis that directly address structural job demands and challenges facing employees. Organizations should rather focus on reducing excessive workload, enhancing managerial support, and redefining work boundaries to create a more balanced and sustainable work environment. Such structural interventions are likely to yield more substantive improvements in employee wellbeing than initiatives aimed solely at promoting individual coping strategies and resourcefulness.

This recommendation aligns with Van Zyl et al. (2024a) who argued that historically there was a shift from occupational health approaches to wellbeing (which focused on working conditions) toward psychological approaches emphasizing individual adaptation. Our findings suggest that during crises, this pendulum may need to swing back toward structural or organizational interventions, rather than forcing individuals to cope with the changes on their own. As Johns (2018) argues, contextualization is crucial for understanding organizational behavior, yet the JD-R model's generalized framework may insufficiently account for the unique contextual features of crisis situations.

In essence, organizations should therefore implement policies that directly address the structural determinants of work-home interference. These might include clearer delineation of work hours during remote work, realistic workload calibration accounting for crisis conditions, formalized communication protocols that respect personal boundaries, and explicit recognition that job security should not entail expectation creep regarding availability and task absorption. These policies are particularly important during periods of organizational disruption, when informal norms may inadequately protect employee wellbeing.

Additionally, our findings suggest that organizational interventions should target the specific job characteristics most strongly associated with negative outcomes during crises. Rather than broadly promoting strengths use or job crafting as universal panaceas, organizations might benefit from tailored interventions addressing specific demand-resource imbalances. This approach aligns with Nielsen et al.'s (2017) recommendation for context-specific interventions rather than one-size-fits-all wellbeing programs.

Furthermore, organizations should recognize that the responsibility for maintaining work-home boundaries should not rest solely with employees. Boundary management is both an individual and organizational responsibility, particularly during crises that fundamentally disrupt established boundary-maintenance practices. Our findings that individual regulatory strategies had limited moderating effects suggest that organizational policies may need to play a more prominent role in boundary preservation during crisis periods.

Finally, our results caution against the uncritical adoption of positive psychological interventions during crisis periods. While strengths use and job crafting have demonstrated benefits in stable organizational contexts (Van Woerkom et al., 2016), their limited moderating effects in our study suggest that their efficacy may be contingent on organizational conditions. Organizations should therefore evaluate the contextual appropriateness of wellbeing interventions rather than implementing them based on generalized efficacy claims. This aligns with Van Zyl et al. (2024a) who called for intervention-context as well as person- intervention fit.

## Limitations and recommendations

Despite the contribution of this study, it is essential to consider its limitations when interpreting the results. First, given the nature of the research question, the sample size was relatively small, thus limiting its generalizability. Further, a large proportion of the participants in this study were employed within the higher education and scientific research sectors. Individuals within these sectors may have already been more accustomed to managing the boundaries between work- and personal life. Further, the nature of work within these environments differs significantly from traditional labor markets, thus making direct comparisons challenging. Second, certain relevant factors such as the number of children, live-in housemates, current working sector, or job function (e.g., employee or manager) were not controlled for in the analyses due to issues with model convergence and limited degrees of freedom. Although excluding these covariates may affect the interpretation of the results, introducing them artificially could potentially bias the findings. Third, the focus of this study was primarily on work-related demands and resources and we did not account for home-related demands and resources. Considering the unique context of the COVID-19 pandemic, the inclusion of home-related factors may have been crucial when assessing the effects of the work-home interference. Finally, the applicability of our results is limited to the specific COVID-19 situation. The assessment of job resources relied on previously developed and validated scales that may not fully capture the unique job resources relevant to the pandemic context. Future research should explore and incorporate these novel job resources to enhance our understanding of the complex dynamics between job characteristics, work-home interference, and wellbeing during crisis situations. Despite these limitations, the extraordinary circumstances presented by the COVID-19 pandemic offer a valuable opportunity to address theoretical gaps and expand existing assumptions within the JDR framework.

These limitations also present opportunities for future research. Our study invites a critical reappraisal of the JDR framework, particularly its assumptions regarding the protective role of personal resources as well as its applicability in times of significant change, uncertainty, and crisis. Future research should investigate the interplay between structural job demands and individual coping strategies under extreme conditions and consider integrating additional contextual variables, such as home-related demands and resources, to better capture the dynamics at play. This expanded perspective may necessitate the development of revised models or extensions of the JDR framework that are specifically tailored to crisis contexts. Further, although it would be impossible to create a comparable situation in the future, there may be an opportunity for a meta-analysis to determine the relationships between these factors across diverse samples, sectors, and labor markets to ensure the broader applicability of the findings. Finally, future research should focus on assessing context-specific job characteristics and control for functional environment factors which may directly impact such. Our limitations also highlight the need for understanding the effect of home-related demands/resources within the JDR framework.

## Conclusion

In conclusion, our findings suggest that the theoretical and practical implications of the JD-R framework require significant recalibration for it to be applicable during times of crisis. Theoretically, the model's assumptions regarding the categorization, stability, and interrelationships of demands and resources appear less tenable during crises. Practically, these theoretical limitations suggest that organizational interventions should prioritize structural changes over individual adaptations during periods of systemic disruption. As organizational crises become increasingly commonplace in a volatile global environment, these recalibrations may be essential for maintaining the JD-R framework's relevance and utility for understanding workplace wellbeing.

Finally, our findings shed light on the importance of considering the challenges contextual factors pose when examining the relationships between job characteristics, personal resources, work-home interference, and psychological wellbeing. When attempting to promote work-life balance, and psychological wellbeing during times of crisis, organizational interventions should directly target work overload, organizational support, and job security, rather than relying solely on individual-level interventions like strengths use and job crafting. Finally, our results call for a reinvestigation of the JD-R model, emphasizing the necessity for organizations to prioritize systemic interventions over personal coping strategies in order to more effectively safeguard employee wellbeing under extreme conditions.

## Data Availability

The datasets presented in this article are not readily available because due to privacy concerns raised by participants during the assessments, the data supporting the study can not be published publicly. Requests to access the datasets should be directed to llewellyn101@gmail.com.

## Footnotes

^1^We controlled for participants English with a self-report item on level of English capability (from 1 = Poor to 5 = Native), and a with attention check questions throughout the survey. If participants self-rated level of English was below 4, and if the participants failed the attention check questions, they were removed from the datasets.

^2^Separate competing measurement models were estimated in Mplus for the four moderating factors in order to determine the best factorial structure for each factor. The results, summarized in Appendix A, showed support for a second order factorial model for overall strengths use (comprised out of two factors: active strengths use and affinity for strengths) and job crafting (comprised out of four first order factors: increasing structural job resources, increasing social job resources, increasing challenging job demands and decreasing hindering job demands). Unidimensional models for PHWI and NHWI were also supported.
