# Correction: Cracks in the JD-R model? The failure of strengths use, job crafting, and home-work spillover to support wellbeing during COVID-19

**DOI:** 10.3389/fpsyg.2025.1719602

**Published:** 2025-10-24

**Authors:** Frontiers Production Office

**Affiliations:** Frontiers Media SA, Lausanne, Switzerland

**Keywords:** job demands, job resources, job crafting, strengths use, work-home interaction, psychological wellbeing, JDR approach

Author Pascale Le Blanc was erroneously included as an author in the published article. The correct author list reads:

Llewellyn Ellardus van Zyl^1,2^^*^, Menno A. Cornelisse^3^ and Sebastiaan Rothmann Sr.^1^

The Author Contributions Statement has been corrected to read:

LvZ: Conceptualization, Data curation, Formal analysis, Investigation, Methodology, Project administration, Resources, Software, Supervision, Validation, Visualization, Writing – original draft, Writing – review & editing. MC: Conceptualization, Data curation, Formal analysis, Investigation, Visualization, Writing – original draft, Writing – review & editing. SR: Conceptualization, Supervision, Resources, Writing – review & editing.

The original version of this article has been updated.

