# Supplementary material for: Cracks in the JD-R model? The failure of strengths use, job crafting, and home-work spillover to support wellbeing during COVID-19
Source: Front Psychol. 2025 Jun 12;16:1532083. doi: 10.3389/fpsyg.2025.1532083 (PMC12199280; doi:10.3389/fpsyg.2025.1532083)
Supplement: Supplementary file 1 [file Supplementary_file_1.pdf]

Appendix A. Descriptive Statistics, reliability and Pearson correlations

No	Construct	x̄	σ	Skw	Ku	Shapiro-Wilk Test		Reliability		Correlation Coefficients																	
						E	*p*	*α*	ω	1	2	3	4	5	6	7	8	9	10	11	12	13	14	15	16	17	18
Process Model Factors																											
1	Work Overload	3.82	0.72	-0.59	0.53	0.96	< .001	0.81	0.81	—																	
2	Organizational Support	3.83	0.74	-0.38	-0.39	0.96	< .001	0.76	0.77	0.16	—																
3	Job Security	3.57	1.25	-0.59	-0.78	0.90	< .001	0.96	0.96	**0.02**	0.20	—															
4	Growth Opportunities	4.37	0.99	-0.31	0.15	0.96	< .001	0.93	0.93	-0.10	0.46	0.18	—														
5	Advancement	3.66	0.91	-0.62	-0.07	0.95	< .001	0.83	0.83	**-0.06**	0.33	0.34	0.22	—													
6	Negative Work-Home Interference	2.90	0.88	0.06	-0.44	0.99	< .001	0.86	0.86	0.54	**-0.07**	-0.22	-0.13	-0.21	—												
7	Positive Work-Home Interference	3.07	0.78	-0.19	-0.25	0.98	< .001	0.73	0.76	**-0.03**	0.29	-0.16	0.16	**0.04**	-0.09	—											
8	Intrinsic Motivation	4.89	1.43	-0.53	-0.01	0.96	< .001	0.90	0.91	0.15	0.60	0.17	0.35	0.30	**-0.05**	0.24	—										
9	Psychological Wellbeing	4.28	0.99	-0.47	-0.13	0.98	< .001	0.86	0.87	**0.05**	0.43	0.19	0.31	0.19	-0.18	0.28	0.46	—									
Moderators																											
10	Strengths Use	5.54	0.86	-0.81	1.14	0.96	< .001	0.94	0.94	0.13	0.48	0.19	0.34	0.20	-0.09	0.31	0.55	0.62	—								
11	Affinity for Strengths	5.56	0.86	-0.81	1.09	0.96	< .001	0.86	0.86	0.13	0.49	0.21	0.33	0.20	-0.10	0.30	0.58	0.55	0.94	—							
12	Active Strengths Use	5.52	0.93	-0.90	1.39	0.95	< .001	0.92	0.92	0.12	0.45	0.17	0.32	0.18	**-0.08**	0.29	0.49	0.62	0.97	0.83	—						
13	Job Crafting	3.39	0.46	-0.03	0.65	0.99	0.027	0.79	0.79	**0.05**	0.27	-0.22	0.17	**0.05**	**-0.05**	0.39	0.30	0.34	0.34	0.31	0.34	—					
14	Increasing Structural Resources	4.06	0.66	-0.93	1.53	0.93	< .001	0.83	0.83	0.13	0.43	0.04	0.24	0.13	**-0.03**	0.36	0.48	0.49	0.52	0.49	0.51	0.60	—				
15	Decreasing Hindering Demands	2.88	0.81	0.05	-0.21	0.99	0.013	0.83	0.83	-0.11	-0.20	-0.30	-0.12	-0.10	**-0.02**	0.11	-0.27	**-0.04**	-0.12	-0.13	-0.11	0.49	-0.10	—			
16	Social Job Resources	3.05	0.86	-0.41	-0.23	0.97	< .001	0.82	0.82	**0.01**	0.16	-0.18	0.13	**0.05**	-0.10	0.19	0.19	0.10	**0.08**	**0.07**	**0.08**	0.68	0.21	0.13	—		
17	Increasing Challenging Demands	3.69	0.75	-0.50	0.30	0.97	< .001	0.81	0.81	0.16	0.39	**0.01**	0.22	**0.08**	**0.05**	0.33	0.47	0.39	0.49	0.45	0.48	0.62	0.53	-0.10	0.22	—	
18	Negative Home-Work Interference	1.96	0.79	0.88	0.65	0.92	< .001	0.85	0.85	0.12	-0.18	-0.41	-0.18	-0.24	0.45	**0.02**	-0.17	-0.30	-0.26	-0.23	-0.25	**0.03**	-0.17	0.21	**0.06**	-0.11	—
19	Positive Home-Work Interference	2.86	1.03	-0.11	-0.78	0.97	< .001	0.88	0.89	**-0.06**	0.14	-0.27	0.09	**-0.03**	**-0.04**	0.65	**0.07**	0.15	0.19	0.21	0.16	0.36	0.25	0.22	0.15	0.22	**0.08**

Bold = Not statistically significant at p<0.05; x̄ = Mean; σ = Standard Deviation; Skw = Skewness; Ku = Kurtosis; ω = McDonald's Omega; α = Cronbach's alpha; E =Estimate

Descriptive statistics (means, standard deviations, skewness, kurtosis, Shapiro-Wilk estimate), Pearson's point-estimate correlations and McDonald's Omega / Cronbach Alphas were computed to determine multivariate normality, to estimate the reliability of the various instruments and to determine the relationships between the factors. Multivariate normality was established if the Skewness and Kurtosis did not exceed +2/-2 and of the Shapiro-Wilk test was significant and above 0.90 (Kim, 2013). The reliability of the instruments was estimated at both the lower-bound (Cronbach Alpha >0.70) and upper-bound (McDonald's Omega > 0.70; Hayes & Coutts, 2020) thresholds. Pearson correlation coefficients were used to explore the relationships between the factors, with the statistical significance set at 95% ($p \leq 0.05$). The descriptive statistics, upper- and lower-bound reliability estimates, and Pearson correlation coefficients are summarised in Appendix A. Table 3 indicates that the data was relatively normally distributed (Skewness/Kurtosis between +2 and -2; Shapiro-Wilk Estimates > 0.90 and p<0.05) and that was reliable at the lower- (Cronbach Alpha > 0.70) and upper bound limits (McDonald's ω > 0.70) (Wang & Wang, 2020). The Pearson correlations coefficients showed statistically significant relationships amongst *most* of the variables ($p < 0.05$).

Appendix B. Competing Measurement Models for Moderating Factors

No	Model	χ^2	*df*	CFI	TLI	RMSEA		SRMR	AIC	BIC	aBIC	Meets Goodness of Fit Criteria	Meets Measurement Quality Criteria
Strengths Use													
1	Unidimensional Model	508.27	77	0.90	0.88	0.11	[.100-.118]	0.05	16732.71	16907.66	16774.36	No	Yes
2	Two First Order Model	422.22	76	0.92	0.90	0.10	[.089-.107]	0.04	16648.66	16827.77	16691.30	Yes	Yes
3	Second Order Model with Two First Order Factors*	356.08	75	0.94	0.92	0.09	[.080-.098]	0.04	16584.52	16767.80	16628.15	Yes	Yes
Job Crafting													
4	Unidimensional Model	2620.28	189	0.41	0.34	0.16	[.158-.169]	0.16	27365.34	27628.29	27428.34	No	No
5	Four First Order Factor Model	697.89	183	0.88	0.86	0.08	[.071-.083]	0.06	25454.96	25742.95	25523.95	No	Yes
6	Second Order Model with Four First Order Factors*	538.29	184	0.91	0.90	0.06	[.057-.070]	0.07	25293.36	25577.18	25361.35	Yes	Yes
Positive Home-Work Interference													
7	Unidimensional Model	10.94	2	0.99	0.98	**0.09**	[.044-.149]	0.02	5437.60	5488.69	5450.60	Yes	Yes
Negative Home-Work Interference													
8	Unidimensional Model	17.89	2	0.98	0.95	0.12	[.075-.179]	0.02	4862.00	4913.09	4875.00	Yes	Yes

χ2 = Chi-square; df = degrees of freedom; TLI = Tucker-Lewis Index; CFI = Comparative Fit Index; RMSEA = Root Mean Square Error of Approximation; SRMR = Standardised Root Mean Square Residual; AIC = Akaike Information Criterion; BIC = Bayes Information Criterion; **Bold** = Non-significant; *two items were permitted to covary
